# Presence of Two Distal and One Mesial Root Canals in Mandibular Second Molars: Report of Four Cases

**Published:** 2014-07-05

**Authors:** Masoud Parirokh, Paul V. Abbott, Mohammad Hosein Yosefi, Hamid Reza Hosseini

**Affiliations:** a Oral and Dental Diseases Research Center, Dental School, Kerman University of Medical Sciences, Kerman, Iran; b Dental School, University of Western Australia, Perth, Australia; c Department of Endodontic, Dental School, Shahid Sadoughi University of Medical Sciences, Yazd, Iran; d Department of Endodontic, Dental School, Kerman University of Medical Sciences, Kerman, Iran

**Keywords:** Anatomic Variation, CBCT, Cone-Beam Computed Tomography, Mandibular Molar, Root Canal Morphology, Root Canal Therapy, Tooth Abnormalities, Tooth Anatomy

## Abstract

Most mandibular second molars have one and two canals in distal and mesial roots, respectively. This report represents four cases of mandibular second molars with a single mesial and two distal root canals with two different canal configurations. After access cavity preparation, two teeth had one distal and two mesial orifices, whereas in the two other teeth one mesial and two distal orifices were found. In the teeth with two mesial canal orifices, the distal root canal and one of root canals with a mesial orifice joined together in the apical part of the root, whereas in the two other teeth with one mesial and two distal canal orifices, three separate canals each with a different apical foramen were detected. Dental practitioners should be aware that despite higher prevalence of one distal and two mesial root canals, the mandibular second molars may also have one mesial and two distal root canals.

## Introduction

Cleaning and shaping of the root canal system is of great importance in root canal treatment (RCT) in order to help achieving maximum success [1]. Every practitioner performing RCT should be aware of the possible root canal configurations of permanent teeth [2-6]. A thorough knowledge of root canal anatomy helps with locating the canal orifices which in turn leads to disinfection of all possible pathways between the pulp space and the periapical tissues. It is essential to disinfect all areas that may harbor microorganisms or necrotic pulp tissues as they may act as a future source for invading bacteria to induce further apical pathosis [1, 7].

Typically, mandibular second molars have either a single root or two roots which may be fused or separate [8-14]. Previous investigations have shown that mandibular second molars may contain one to five root canals [3, 8-14]. Various root canal configurations have been described for these teeth including C-shaped canals, a single root with a single root canal, two roots (one mesial and one distal) each with either a single root canal, two mesial canals and one distal canal, two mesial and two distal root canals, *etc*. [8-18].

Wells and Bernier have reported a mandibular second molar tooth with one mesial and two distal root canals that joined together in the apical part of the distal root [15]. This case report presents four cases with one mesial and two distal root canals with two different root canal configurations.

## Case Report

***Cases 1 and 2-mandibular second molars with one mesial and two distal orifices***

**Figure 1:**
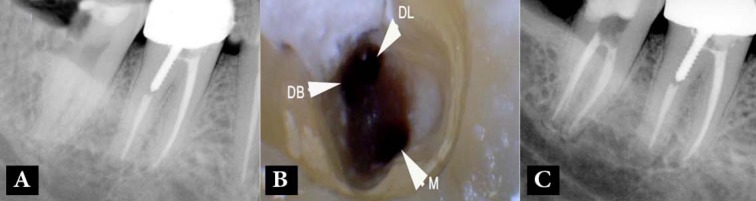
Case-1; *A)* Pre-operative radiograph shows the mandibular second molar with two separate roots; *B)* One mesial (M) and two distal (DB: distobuccal, DL: distolingual) canal orifices; *C)* Post-operative radiograph; two separate distal root canals and one mesial canal

A 36-year old female presented with spontaneous pain in her right mandibular second molar. She had no relevant medical history. Her dental history indicated that the tooth had been restored with an occlusal composite restoration several years prior to the current problem. On examination, caries was noted on the distal aspect of the crown. Pulp sensibility tests were indicative of acute irreversible pulpitis (prolonged pain upon a cold stimulus). A periapical radiograph showed that the tooth had two separate roots with a very small periapical radiolucency surrounding the apex of the mesial root (Figure 1A).

**Figure 2:**
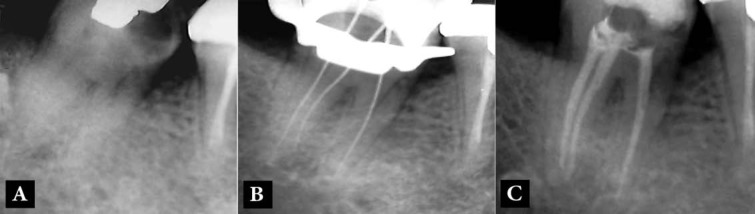
Case-2; *A)* Pre-operative radiograph shows the mandibular second molar with two separate roots; *B)* Working radiograph shows one mesial and two separate distal root canals; *C)* Post-operative radiograph; one mesial and two distal canals can be seen

After administration of local anesthesia with 2% lidocaine containing 1:80000 epinephrine (Darupakhsh, Tehran, Iran), the caries was removed and an access cavity was prepared. Three root canal orifices, *i.e*. one mesial and two distal canals, were located (Figure 1B). The root canals were prepared with Hero 642 rotary instruments (Micro-Mega, Besancon, France), irrigated with 5.25% NaOCl followed by 17% ethylenediaminetetraacetic acid (EDTA, Asia Chemi Teb. Co., Tehran, Iran) and then filled with gutta-percha and AH-26 root canal sealer (Dentsply, De Trey, Konstanz, Germany) (Figure 1C).

The second case was a 35-year old female with no relevant medical history was referred to the postgraduate dental clinic of the Kerman Dental School, Kerman, Iran. The patient was experiencing spontaneous pain stemming from her right mandibular second molar. A periapical radiograph showed mesial caries and two separate roots with no periapical radiolucency (Figure 2A). Cold pulp sensibility test (Endo-Frost; Roeko, Langenau, Germany) induced severe, exaggerated pain but the tooth was not tender to percussion or palpation. The tooth was diagnosed as having acute irreversible pulpitis. After administration of local anesthesia and placing rubber dam, the caries were removed and an access cavity was prepared. Three canal orifices were located. After insertion of endodontic instruments into the root canals, a periapical radiograph was taken and showed three separate root canals (one mesial and two separate distal root canals) (Figure 2B). The root canals were prepared and obturated similar to the first case (Figure 2C).

***Cases 3 and 4- mandibular second molars with two mesial and one distal canal orifices***

**Figure 3:**
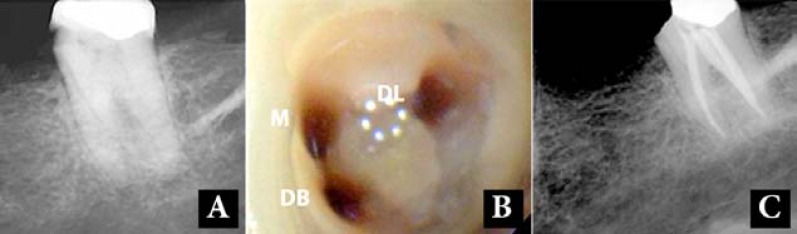
Case-3; *A)* Pre-operative radiograph shows the mandibular second molar. *B)* Two canals in mesial (M and DB) and one in distal (DL) of the chamber floor were detected. *C)* Post-operative radiograph shows that one mesial and two distal canals have been filled

A 48-year old female with no relevant medical history presented with a history of spontaneous lingering pain and sensitivity to cold and hot beverages in her left mandibular second molar. A periapical radiograph showed caries in the mesial aspect (Figure 3A). The tooth was sensitive to cold and heat tests but it was not sensitive to percussion and palpation. The tooth was diagnosed with acute irreversible pulpitis. After administration of 2% lidocaine with 1:80000 epinephrine with mandibular block technique, the caries was removed and the pulp was exposed. An access cavity was made and three separate canal orifices (two mesial and one distal) were detected (Figure 3B). The working length radiograph indicated that the mesiobuccal and the distal root canals joined together and had one apical foramen, whereas the mesiolingual canal was separate and was located in the mesial root. The root canals were prepared with RaCe rotary instruments (FKG Dentaire, La Chaux-de-Fonds, Switzerland) up to #35/0.04, irrigated with 5.25% NaOCl and 17% EDTA, dried and filled with gutta-percha and AH-26 sealer using the lateral condensation technique (Figure 3C).

**Figure 4:**
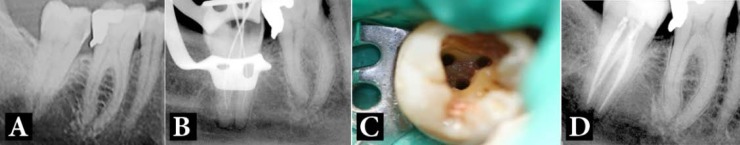
Case-4; *A)* Pre-operative radiograph showing the mandibular second molar; *B)* The working radiograph showing one mesial and two distal root canals; *C)* Detection of two mesial and one distal orifices; *D)* Post-operative radiograph; one mesial and two distal canals have been filled

The last case was a 28-year old female presented with pain in her right mandibular second molar. The tooth had a history of spontaneous pain and prolonged pain associated with cold foods and drinks. The patient’s past dental history indicated that the tooth had been restored with a disto-occlusal amalgam restoration years earlier. The tooth had prolonged pain on cold pulp sensibility test but it had normal responses to heat and percussion. A carious lesion was detected in the mesial aspect of the tooth during the clinical examination and on the periapical radiograph (Figure 4A). After caries removal the pulp was exposed. An access cavity was prepared and three root canal orifices were located; two mesial and one distal orifices (Figure 4B). After placing instruments into the root canals, a periapical radiograph showed that the canal with a mesiobuccal orifice and the distal canal joined together and exited through a single root canal foramen (Figure 4C). The root canals were prepared and filled as outlined earlier for case 3 (Figure 4D).

## Discussion

This report presents four cases of mandibular second molars with one mesial and two distal root canals that represented two different root canal configurations.

One of the “must learn” aspects for practicing root canal treatment is having thorough knowledge of root canal morphology [2, 18, 19]. Several reports have outlined different root canal anatomies for mandibular second molars [3, 4, 8-14, 18]. These reports typically state the percentage of root canals in each root but they do not indicate how many root canal(s) may be expected in the distal root when the mesial root has only one canal [8-14]. Treatment of the first two cases (cases 1 and 2) have an important message to the dental practitioners because most dentists would not expect to detect two separate root canals in the distal root of the mandibular second molars when the mesial root has only one root canal.

Cases 3 and 4 have similar morphology to a case reported by Wells and Bernier [15]. In both cases, despite the presence of one distal and two mesial orifices, the distal root had two root canals, whereas the mesial root had only one root canal. It is very important to recognize that one canal appeared to be in the mesiobuccal part of the tooth (based on the location of its orifice) but it was actually in the distal root. An inexperienced dentist may be unaware of this type of root canal anatomy and this may lead to complications such as ledge formation or perforation if inappropriate instruments (*e.g*. Gates Glidden or Peeso reamer drills) are used before knowing the root canal morphology throughout the entire length of the root. Modern endodontic teaching should encourage detection of the root canal pathways prior to preparation of the canals [20]. In cases 3 and 4, the advantage of this recommendation can be observed. This also emphasizes the advantages of using periapical radiographs in determining the working length of root canals since radiographs provide a visual representation of the pathway of the file and canal. If apex locators are used as the sole means of determining the working length, then the true pathway of the canal will be unknown and treatment errors may result.

The pre-operative radiographic images of three of the cases (case 1, 3, and 4) were not indicative of the complex root canal morphology. The operator’s judgment regarding these teeth was based on clinical investigation and further radiographic images during treatment. Previously reported studies on root canal morphology have mainly used extracted teeth for this purpose which is not feasible in clinical setting. Cone-beam computed tomography (CBCT) may be used to distinguish between single or fused roots of teeth [4, 21-23], but the use of CBCT when periapical radiographs show ordinary root canal anatomy seems unethical due to the unnecessary and excessive exposure to radiation for the patient. In addition, a recent paper of the American Association of Endodontists (AAE) and the American Academy of Oral and Maxillofacial Radiology (AAOMR) does not support routine CBCT for all cases except when complex root canal anatomy is suspected [23]. In the present report, two different root canal configurations have been described. These are different from the common configurations reported for mandibular second molars with one distal and two mesial root canals [8-14].

An interesting finding in this case series is that all four cases were female patients. However, the number of cases is very small and it is not possible to conclude that this type of root canal configuration can be related to the patient’s gender.

## Conclusion

In conclusion, the present report revealed two unusual forms of root canal anatomy of mandibular second molars. Practitioners should be aware of these possible forms and check the access cavity for the presence of all orifices and root canals.
